# Three new *ent*-abietane diterpenoids from the roots of *Euphorbia fischeriana* and their cytotoxicity in human tumor cell lines

**DOI:** 10.1007/s12272-019-01151-y

**Published:** 2019-04-17

**Authors:** Minghui Li, Fang He, Yuan Zhou, Meigui Wang, Pingde Tao, Qingmei Tu, Guanghui Lv, Xintao Chen

**Affiliations:** 1 Department of Pharmacy, Taihe Hospital, Hubei University of Medicine, Shiyan, 442000 People’s Republic of China; 2 Hubei Key Laboratory of Natural Medicinal Chemistry and Resource Evaluation, School of Pharmacy, Tongji Medical College, Huazhong University of Science and Technology, Wuhan, 430030 People’s Republic of China

**Keywords:** *ent*-Abietane diterpenoids, *Euphorbia fischeriana*, Structural elucidation, Cytotoxicity

## Abstract

**Abstract:**

Three new *ent*-abietane diterpenoids, termed fischerianoids A–C (**1**–**3**), were isolated and identified from the ethyl acetate extracts of roots of the medicinally valuable plant *Euphorbia fischeriana*. The planar and relative structures of **1**–**3** were established via high-resolution electrospray ionisation mass spectrometry and one- and two-dimensional nuclear magnetic resonance spectroscopic analyses, and the absolute configuration of **1** was further established via X-ray crystallography experiment. Compounds **1**–**3** showed selective inhibitory potency against certain human tumor cell lines with IC_50_ values ranging from 8.50 ± 0.13 to 35.52 ± 0.08 μM.

**Graphical abstract:**

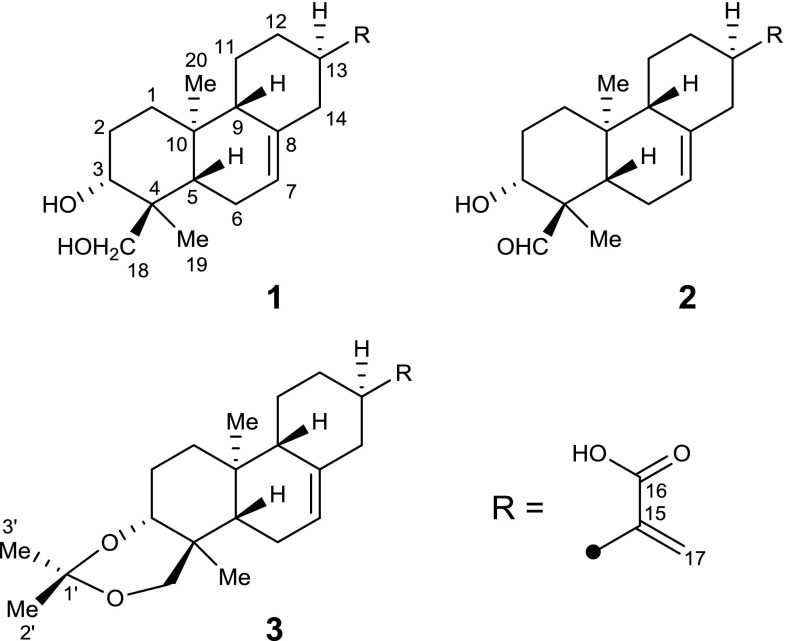

**Electronic supplementary material:**

The online version of this article (10.1007/s12272-019-01151-y) contains supplementary material, which is available to authorized users.

## Introduction

*Euphorbia fischeriana* Steud, a perennial herbaceous plant of the Euphorbiaceae family, is widely distributed in northeastern mainland China (Wang et al. 2012). The roots of *E. fischeriana*, also named “Lang-du”, are widely used in folk medicine for the treatment of edema, ascites, and cancer (Pan et al. 2011). More importantly, an extract of *E. fischeriana* is employed as the main ingredient of the drug “Jie-He-Ling”, which is used in clinical practice to treat lymphoid and pulmonary tuberculosis (Li et al. 2016). Previous chemical investigations have suggested that the roots of this plant are a rich source of diterpenoids, triterpenoids, steroids, polysaccharides, and acetophenone derivatives, all of which have diverse biological properties, such as antibacterial, anti-inflammatory, anti-tuberculosis, and cytotoxic activities (Wang et al. 2017). Of these diterpenoids, jolkinolides A and B exhibit potential cytotoxic activities against Ehrlich ascites, sarcoma 180, and HeLa cells; meanwhile 17-hydroxyjolkinolide B is a promising anti-cancer drug candidate as it potently inhibits signal transducer and activator of transcription 3 signaling (Pan et al. 2011). Prostratin (12-deoxyphorbol-13-acetate) is a 12-deoxytigliane diterpenoid, which acts as a protein kinase C activator and is potentially used to treat latent human immunodeficiency virus. The challenge of its semi synthesis and total chemical synthesis have been of great interest to chemists in recent years (Tong et al. 2018). Although its mechanism of action has not been completely investigated, prostratin has been advanced into preclinical trials (Wang et al. 2006).

**Fig. 1:**
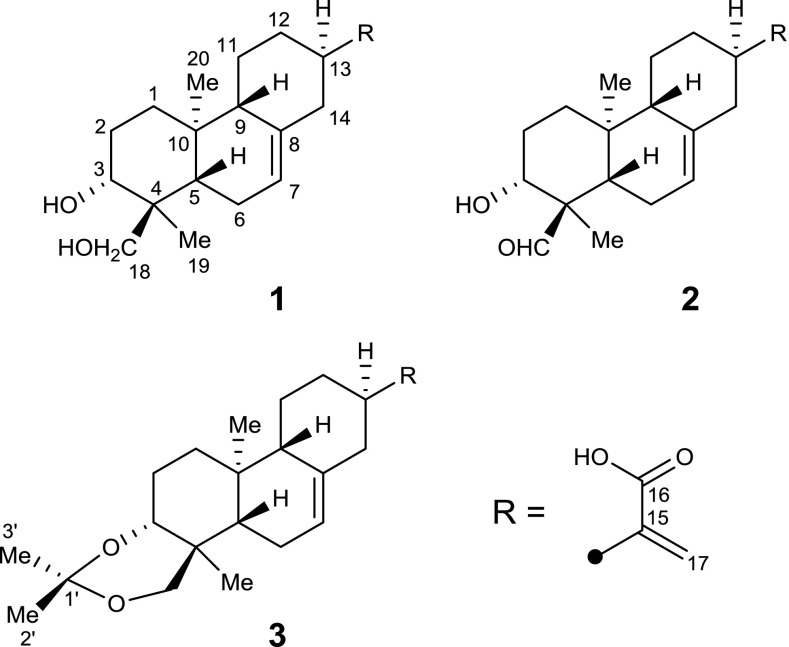
Chemical structures of compounds **1**–**3**

As part of our ongoing program for discovering structurally interesting and bioactive metabolites from plants growing in the Wudang Mountain area, the medicinally valuable plant *E. fischeriana* was chemically investigated, which led to the isolation and identification of three new ent-abietane diterpenoids, termed fischerianoids A–C (**1**–**3**). Here, we report the isolation, structural elucidation, and cytotoxic activity of these compounds (Fig. 1).

## Materials and methods

### General experimental procedures

Optical rotations were collected on the JASCO P2000 automatic digital polarimeter. Ultraviolet visible spectra were collected on a Hewlett-Packard HP-845 UV–VIS spectrophotometer. FT-IR spectra were recorded on the Bruker Vertex 70 instrument. 1D and 2D nuclear magnetic resonance (NMR) spectra were measured on the Bruker AM-400 spectrometer, with pyridine-*d*_5_ as the solvent and tetramethylsilane (TMS) as the internal standard. The chemical shifts were reported in *δ* (ppm) units relative to the TMS signal and coupling constants (*J*) in Hz. High-resolution electrospray ionisation mass spectrometry (HRESIMS) data were collected in the positive ion mode on the Thermo Fisher LC-LTQ-Orbitrap XL spectrometer. Column chromatography separations were performed on a silica gel (200–300 mesh; Qingdao Haiyang Chemical Co. Ltd, Qingdao, China), ODS column (50 mesh, AA12S50; YMC; Merck Co. Ltd., Germany), and Sephadex LH-20 liquid chromatography media (Pharmacia, Peapack, NJ, USA). Semi-preparative high-performance liquid chromatography (HPLC) analysis was performed with the Agilent 1100 quaternary system using the reversed-phased C_18_ column (5 μm, 10 × 250 mm). Thin layer chromatography was carried out with pre-coated silica gel 60 F_254_ (0.25 mm; Merck, Kenilworth, NJ, USA) plates, and spots were visualized by spraying heated silica gel plates with 10% sulfuric acid in ethanol.

### Plant material

The roots of *E. fischeriana* were collected from the Wudang Mountain area of Hubei Province of China in September 2016. The voucher specimen (20160925) was deposited in Hubei University of Medicine (Shiyan, China).

### Extraction and isolation

The air-dried roots of *E. fischeriana* (4.0 kg) were chopped and extracted with 80% acetone (3 × 5 L) at ambient temperature (298 K). The combined extracts were concentrated under reduced pressure to yield a crude residue (140 g), which was suspended in water (2.0 L) followed by successive partitions with ethyl acetate (EtOAc) (3 × 1.5 L) and *n*-BuOH (3 × 2.0 L). The EtOAc extracts (45 g) were subjected to a silica gel column chromatography eluted with CH_2_Cl_2_–acetone (1:0–1:1, v/v) to afford six fractions, Fr.1–Fr.6. Then Fr.3 (12 g) was loaded onto an RP-C_18_ column with stepwise MeOH–H_2_O (30:70–100:0, v/v) to yield five fractions (Fr.3.1–Fr.3.5). Fr.3.2 (3.3 g) was purified by silica gel column chromatography (petroleum ether–EtOAc, 2:1–1:2) and repeated semi-preparative HPLC analysis using MeOH–H_2_O (45:55, 2.5 mL/min) as eluent to provide compound **3** (7.6 mg, *t*_R_ = 35 min). Fr.4 (15 g) was purified by an RP-C_18_ CC using MeOH–H_2_O (30:70–70:30, v/v), a silica gel CC eluted with CH_2_Cl_2_–MeOH (60:1–10:1, v/v), and repeated semi-preparative HPLC using MeOH–H_2_O (56:44, 2.5 mL/min) to afford compounds **1** (21.1 mg, *t*_R_ = 17 min) and **2** (8.4 mg *t*_R_ = 22 min).

#### Fischerianoid A (**1**)

Colorless crystals; [*α*]23 D: +13.5 (*c* 0.2, MeOH); UV (MeOH) *λ*_max_ (log *ε*): 204 (4.18) nm; IR (*ν*_max_): 3429, 1693, 1628, 1551, 1384, 1055, 584 cm^−1^; for ^1^H- and ^13^C-NMR spectroscopic data, see Table 1; HRESIMS *m/z* 333.2070 ([M−H]^−^, C_20_H_29_O_4_, calcd 333.2071).

**Table 1:** ^1^H and ^13^C NMR spectrographic data for compounds **1**–**3** (*δ* in ppm, *J* in Hz)

No.	**1** ^a^		**2** ^a^		**3** ^a^	
	*δ*_H_	*δ*_C_	*δ*_H_	*δ*_C_	*δ*_H_	*δ*_C_
1	1.24 m; 1.82 m	38.4 t	1.21 m; 1.82 m	38.1 t	1.16 m; 1.76 m	38.5 t
2	1.94 m	28.2 t	1.91 m	27.7 t	1.55 m; 1.69 m	24.8 t
3	4.27 m	73.7 d	4.12 dd (4.6, 10.9)	72.7 d	3.64 dd (3.5, 11.6)	77.8 d
4	–	43.4 s	–	56.1 s	–	37.2 s
5	1.93 m	43.3 d	1.71 m	42.5 d	1.09 dd (4.4, 12.3)	46.2 d
6	2.03 m	23.7 t	1.47 m; 2.01 m	25.0 t	1.51 m; 1.87 m	22.9 t
7	5.42 s	121.3 d	5.34 m	120.3 d	5.39 d (3.3)	120.5 d
8	–	137.4 s	–	137.8 s	–	137.8 s
9	1.75 m	52.8 d	1.75 m	52.4 d	1.71 m	52.8 d
10	–	35.6 s	–	34.7 s	–	35.8 s
11	1.23 m; 1.76 m	26.1 t	1.19 m; 1.74 m	26.1 t	1.19 m; 1.69 m	25.9 t
12	1.28 m; 2.04 m	32.5 t	1.31 m; 2.05 m	32.3 t	1.32 m; 2.05 m	32.3 t
13	2.80 m	40.0 d	2.77 m	40.0 d	2.78 m	40.0 d
14	2.02 m; 2.66 m	41.9 t	2.04 m; 2.65 m	41.7 t	2.07 m; 2.69 m	41.9 t
15	–	147.8 s	–	147.6 s	–	147.8 s
16	–	170.1 s	–	170.1 s	–	170.2 s
17	5.64 s; 6.54 s	122.1 t	5.64 s; 6.55 s	122.2 t	5.65 s; 6.56 s	122.2 t
18	3.68 d (10.6); 4.14 d (10.6)	67.7 t	9.62 s	207.2 d	3.47 d (10.5); 3.58 d (10.5)	72.7 t
19	1.16 s	13.4 q	1.44 s	10.1 q	1.25 s	13.3 q
20	0.93 s	16.2 q	0.84 s	15.9 q	0.83 s	16.2 q
1′	–	–	–	–	–	99.4 s
2′	–	–	–	–	1.53 s	19.8 q
3′	–	–	–	–	1.55 s	30.6 q

^a^Recorded in pyridine-*d*_5_, 400 MHz for *δ*_H_, 100 MHz for *δ*_C_

#### Fischerianoid B (**2**)

White powders; [*α*]23 D: –15.2 (*c* 0.2, MeOH); UV (MeOH) *λ*_max_ (log *ε*): 203 (4.12) nm; IR (*ν*_max_): 3418, 2934, 1716, 1630, 1383, 1221, 1056, and 655 cm^−1^; for ^1^H- and ^13^C-NMR spectroscopic data, see Table 1; HRESIMS *m/z* 331.1915 ([M−H]^−^, C_20_H_27_O_4_, calcd 331.1915).

#### Fischerianoid C (**3**)

White powders; [*α*]23 D: +25.8 (*c* 0.2, MeOH); UV (MeOH) *λ*_max_ (log *ε*): 205 (3.89) nm; IR (*ν*_max_): 3421, 2924, 2854, 1677, 1619, 1442, 1381, 1259, 1207, 1156, 1100, 1034, and 864 cm^−1^; for ^1^H- and ^13^C-NMR spectroscopic data, see Table 1; HRESIMS *m/z* 373.2380 ([M−H]^−^, C_23_H_33_O_4_, calcd 373.2384).

### X-ray crystallography analysis

Crystals of **1** were obtained from a mixed solution (methanol/chloroform/water = 4:4:1). The intensity data for **1** were collected at 100 K on a Bruker APEX DUO diffractometer equipped with an APEX II CCD using Cu K*α* radiation. Data reduction and cell refinement were conducted with Bruker SAINT (Sheldrick and Schneider 1997). The structures of the compounds were solved by direct methods using SHELXS-97, different Fournier techniques were used to expand the data, and the structures were refined by fullmatrix least-squares calculations. Hydrogen atoms were placed at calculated positions and non-hydrogen atoms were anisotropically refined. Crystallographic data for the structure have been deposited in the Cambridge Crystallographic Data Center with deposition (No. CCDC 1880988 for **1**). Copies of the data can be obtained free of charge from the CCDC, 12 Union Road, Cambridge CB 1EZ, UK [fax: Int. +44(0) (1223) 336 033); e-mail: deposit@ccdc.cam.ac.uk].

Crystallographic data for compound **1**: C_20_H_30_O_4_, *M* = 334.44, monoclinic, *a* = 10.4886(5) Å, *b* = 7.3402(4) Å, *c* = 11.3529(6) Å, *α* = 90.00°, *β* = 99.013(2)°, *γ* = 90.00°, *V* = 863.25(8) Å^3^, *T* = 100(2) K, space group *P*21, *Z* = 2, *μ*(Cu Kα) = 0.703 mm^−1^, 5155 reflections measured, 2536 independent reflections (*R*_*int*_ = 0.0595). The final *R*_*1*_ values were 0.1336 (*I* > 2*σ*(*I*)). The final *wR*(*F*^2^) values were 0.3012 (*I* > 2*σ*(*I*)). The final *R*_*1*_ values were 0.1337 (all of the data). The final *wR*(*F*^2^) values were 0.3016 (all of the data). The goodness of fit on *F*^2^ was 1.499. Flack parameter = 0.1(4).

## Biological evaluation

### Cytotoxicity assays

Six human tumor cell lines, HL-60, SMMC-7721, MM-231, A-549, HEP3B, and SW-480, along with one noncancerous cell line, the human normal colonic epithelial cell NCM460, were used in the cytotoxicity assay (Li et al. 2018). All of the cells were cultured in Dulbecco’s Modified Eagle’s Medium or RPMI-1640 medium (HyClone, Logan, UT, USA) supplemented with 10% fetal bovine serum (HyClone) at 37 °C in a humidified atmosphere with 5% CO_2_. The MTT assay was conducted to measure cell survival. In brief, a suspension (100 μL) of adherent cells was seeded into 96-well culture plates and allowed to adhere for 12 h before addition of the tested compounds. The suspended cells were seeded at a density of 1 × 10^5^ cells/mL immediately before drug additions. Each tumor cell line was exposed for 48 h in triplicate to the tested compounds at concentrations of 0.0625, 0.32, 1.6, 8, and 40 μM, and cisplatin (DDP; Sigma, St. Louis, MO, USA) was selected as the positive control. After incubation, culture supernatants were removed and exchanged with medium containing MTT (0.5 mg/mL). Then the cells were incubated at 37 °C for 4 h in the dark, followed by the removal of the medium and addition of dimethyl sulfoxide (100 μL). The absorbance at 570 nm was measured, and the data were expressed as the average of three replicates of absorbance in treated versus control cells.

## Results

### Isolation and structural elucidation of diterpenoids (1–3)

Fischerianoid A (**1**) was obtained as white powders, and had a molecular formula of C_20_H_30_O_4_ based on its HRESIMS data at *m/z* 333.2070 ([M−H]^−^, calcd 333.2071), corresponding to six degrees of unsaturation. Detailed interpretation of the ^1^H- and ^13^C- NMR and HSQC spectra disclosed the obvious signals assigned as follows: two singlet methyls [*δ*_H_ 1.16/*δ*_C_ 13.4 (C-19); *δ*_H_ 0.93/*δ*_C_ 16.2 (C-20)], eight methylenes [*δ*_H_ 1.24 and 1.82/*δ*_C_ 38.4 (C-1); *δ*_H_ 1.94/*δ*_C_ 28.2 (C-2); *δ*_H_ 2.03/*δ*_C_ 23.7 (C-6); *δ*_H_ 1.23 and 1.76/*δ*_C_ 26.1 (C-11); *δ*_H_ 1.28 and 2.04/*δ*_C_ 32.5 (C-12); *δ*_H_ 2.02 and 2.66/*δ*_C_ 41.9 (C-14); *δ*_H_ 5.64 and 6.54/*δ*_C_ 122.1 (C-17); *δ*_H_ 3.68 and 4.14/*δ*_C_ 67.7 (C-18)], five methines [*δ*_H_ 4.27/*δ*_C_ 73.7 (C-3); *δ*_H_ 1.93/*δ*_C_ 43.3 (C-5); *δ*_H_ 5.42/*δ*_C_ 121.3 (C-7); *δ*_H_ 1.75/*δ*_C_ 52.8 (C-9); *δ*_H_ 2.80/*δ*_C_ 40.0 (C-13)], and five quaternary carbons [*δ*_C_ 43.4 (C-4); *δ*_C_ 137.4 (C-8); *δ*_C_ 35.6 (C-10); *δ*_C_ 147.8 (C-15); *δ*_C_ 170.1 (C-16)]. Among them, two double bonds and one carboxyl group occupied three of six degrees of unsaturation, suggesting that this compound had a tricyclic ring system.

**Fig. 2:**
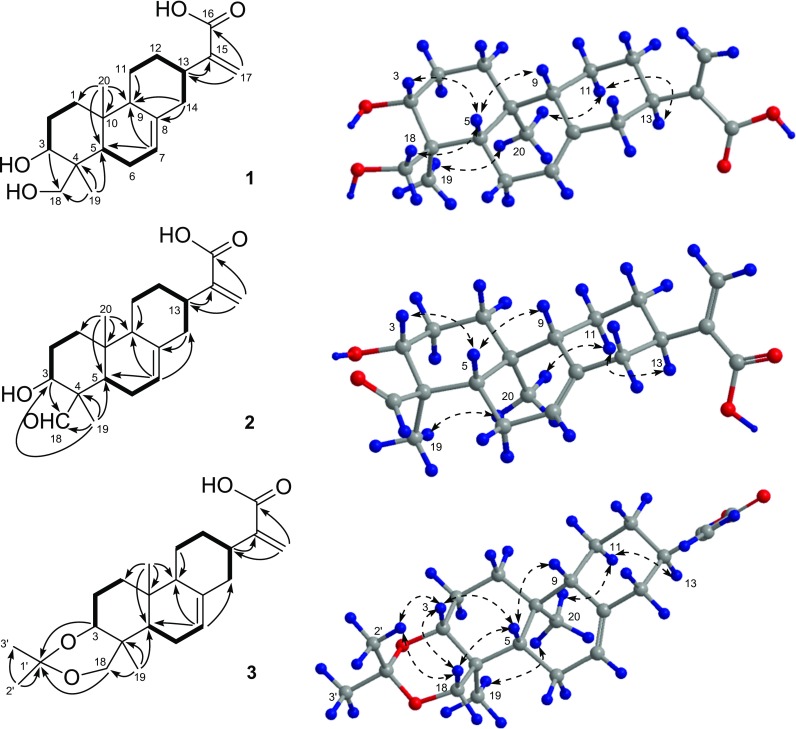
Key ^1^H–^1^H COSY (bold lines), HMBC (solid arrows), and NOESY (dashed arrows) correlations of **1**–**3**

Detailed analyses of the 2D NMR spectra (Fig. 2) of **1** suggested that it was a structural analogue of *ent*-abieta-7,15(17)-diene-3*β*,16,18-triol (Niu et al. 2003), identified as an *ent*-abietane diterpenoid, with the difference that the oxygenated methylene was replaced by a carboxyl group in **1**. In the HMBC spectrum, the correlations arising from H-3 to C-18 and from Me-19 to C-3, C-4, C-5, and C-18 suggested that C-3 and C-18 were both substituted by the hydroxyl groups. Additionally, the HMBC correlations from H-7 to C-5 and C-9 and from H_2_-14 to C-8 suggested that a double bond was positioned between C-7 and C-8. The HMBC correlations arising from H_2_-17 to C-13 and C-16 and from H-13 to C-15 suggested that an exocyclic double bond was positioned between C-15 and C-17 and a carboxy group was located at C-16. Therefore, the planar structure of **1** was determined.

**Fig. 3:**
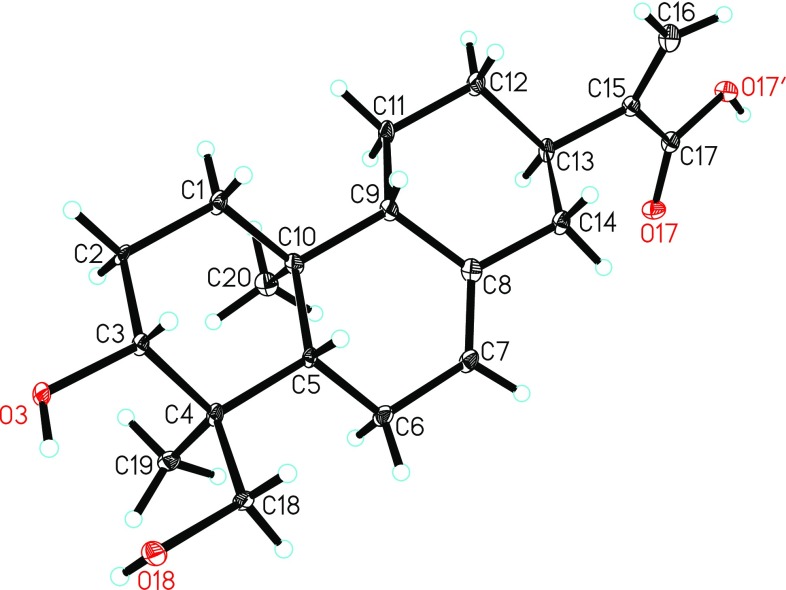
ORTEP drawing of compound **1**

The relative configuration of **1** was determined by the NOESY experiment (Fig. 2). The correlations from H-5 to H-3, H-9, and H_2_-18 and cross-peaks of Me-19/Me-20/H-11*α* (*δ*_H_ 1.23)/H-13 indicated that H-13, Me-19, and Me-20 were all *α*-oriented, on the contrary, H-3, H-5, H-9, and CH_2_(OH)-18 were all *β*-oriented. Accordingly, the relative configuration of **1** was defined, which was consistent with that of *ent*-abieta-7,15(17)-diene-3*β*,16,18-triol. To further determine its absolute configuration, the crystallographic approach was considered. Fortunately, after repeated recrystallization by various two-phase or three-phase solvents, a suitable crystal of **1** was obtained from methanol/chloroform/water (4:4:1) at room temperature, which was then analyzed by a single-crystal X-ray diffraction experiment using Cu K*α* (Fig. 3). On the basis of a Flack parameter of 0.1(4) (CCDC 1880988) (Flack and Bernardinelli 1999), its absolute configuration was determined to be 3*R*,4*S*,5*S*,9*S*,10*S*,13*R* and *Z*-geometry of the double bond between C-7 and C-8.

Fischerianoid B (**2**) was obtained as white powders, and possessed a molecular formula of C_20_H_28_O_4_ based on the HRESIMS analysis at *m/z* 331.1915 ([M−H]^−^, calcd 331.1915), which was two hydrogen atoms less than **1**. Detailed comparison of the 1D NMR data (Table 1) of **2** with those of **1** suggested that both compounds were structural analogues. The only difference was that one aldehyde group at *δ*_C_ 207.2 (C-18, compound **2**) replaced one oxygenated methylene group at *δ*_C_ 67.7 (C-18, compound **1**). This deduction was further confirmed by the HMBC correlations (Fig. 2) arising from Me-19 to C-3, C-4, C-5, and C-18 and from H-3 to C-18. Accordingly, the structure of **2** was determined.

The molecular formula C_23_H_34_O_4_ of fischerianoid C (**3**) was deduced from the HRESIMS data at *m/z* 373.2380 ([M−H]^−^, calcd 373.2384) and ^13^C NMR data, which corresponded to seven degrees of unsaturation. A side-by-side comparison of the 1D NMR data (Table 1) of **3** with those of **1** indicated that three additional carbon signals for the acetonide group [CH_3_-2′ (*δ*_H_ 1.53/*δ*_C_ 19.8), CH_3_-3′ (*δ*_H_ 1.55/*δ*_C_ 30.6), and C-1′ (*δ*_C_ 99.4)] were present, suggesting that **3** was an acetonide derivative of **1**. This conclusion was further supported by one more degree of unsaturation required in the molecule of **3** and the HMBC correlations (Fig. 2) arising from H-3 and H_2_-18 to C-1′ and from Me-2′ to C-1′ and C-3′. Accordingly, the structure of **3** was determined. Remarkably, compound **3** was most possibly an artifact of compound **1** generated during the extraction process.

The relative configurations of compounds **2** and **3** were determined by analysis of NOESY data as shown in Fig. 2. Since compounds **1**–**3** shared the same backbones, the relative and absolute configurations of **2** and **3** were analogous to those of compound **1**, whose absolute configuration was determined by X-ray crystallographic analysis.

Considering that the *ent*-abietane diterpenoids reported from the roots of *Euphorbia fischeriana* showed significant cytotoxicity (Wang et al. 2006), compounds **1**–**3** were evaluated for the cytotoxicity (Table 2) against several tumor cell lines, including HL-60 (acute leukemia), MM-231 (breast cancer), SMMC-7721 (hepatic cancer), A-549 (lung cancer), HEP3B (hepatic cancer), SW-480 (colon cancer), and one normal colonic epithelial cell NCM460. As a result, compounds **1**–**3** showed selective inhibitory potency against certain human tumor cell lines with IC_50_ values ranging from 8.50 ± 0.13 to 35.52 ± 0.08 μM, with no obvious cytotoxicity to the normal cell NCM460.

**Table 2:** Cytotoxic activity of **1**–**3** against several human tumor cell lines

No.	IC_50_ (*µ*M)						
	HL-60	MM-231	SMMC-7721	A-549	HEP3B	SW-480	NCM460
**1**	> 40	12.10 ± 0.21	32.48 ± 0.13	> 40	15.95 ± 0.15	> 40	> 40
**2**	28.78 ± 0.17	9.12 ± 0.21	> 40	> 40	8.50 ± 0.13	35.52 ± 0.08	> 40
**3**	> 40	25.45 ± 0.12	> 40	> 40	27.34 ± 0.05	> 40	> 40
Cisplatin^a^	1.60 ± 0.10	3.82 ± 0.23	2.78 ± 0.15	2.81 ± 0.35	2.97 ± 0.21	1.45 ± 0.12	0.87 ± 0.10

^a^Cisplatin was used as the positive control

## Discussion

Previous phytochemical investigations on *E. fischeriana* have demonstrated that ent-abietane diterpenoids account for the characteristic constituents of this species (Wang et al. 2006; Lee et al. 2016; Wang et al. 2017). In this study, three new *ent*-abietane diterpenoids, termed fischerianoids A–C (**1**–**3**), were isolated and identified from the EtOAc extracts of the roots of *E. fischeriana*. The planar and relative structures of **1**–**3** were established via extensive spectroscopic data (HRESIMS and 1D and 2D NMR) analyses, and the absolute configuration of **1** was further established via X-ray crystallography experiment with Cu K*α* radiation. Compound **3** was characterized by a rare acetonide motif but most possibly an artifact of compound **1** generated during the extraction process. Additionally, compounds **1**–**3** showed selective inhibitory potency against certain human tumor cell lines with IC_50_ values ranging from 8.50 ± 0.13 to 35.52 ± 0.08 μM, with no obvious cytotoxicity to the normal cell NCM460. These findings not only enrich the new numbers of ent-abietane diterpenoids, but also extend the pharmaceutical usage of ent-abietane diterpenoids to cytotoxic activity. More importantly, they may play important roles in the chemotaxonomic significance of *E. fischeriana* as well as the genus Euphorbia.

## Electronic supplementary material

Below is the link to the electronic supplementary material.

Supplementary material 1 (DOC 1380 kb)
