# Supplementary material for: Three new ent-abietane diterpenoids from the roots of Euphorbia fischeriana and their cytotoxicity in human tumor cell lines
Source: Arch Pharm Res. 2019 Apr 17;42(6):512–8. doi: 10.1007/s12272-019-01151-y (PMC6562045; doi:10.1007/s12272-019-01151-y)
Supplement: Supplementary file 1 — Supplementary material 1 (DOC 1380 kb) [file 12272_2019_1151_MOESM1_ESM.doc]

# Three new *ent*-abietane diterpenoids from the roots of *Euphorbia fischeriana* and their cytotoxicity in human tumor cell lines

Minghui Li,^1,†^ Fang He,^1,†^ Yuan Zhou,^1^ Meigui Wang,^1^ Pingde Tao,^1^ Qingmei Tu,^1^ Guanghui Lv,^1^,* and Xintao Chen^1,2^,*

*^1^ Department of Pharmacy, Taihe Hospital, Hubei University of Medicine, Shiyan 442000, People’s Republic of China*

*^2^ Hubei Key Laboratory of Natural Medicinal Chemistry and Resource Evaluation, School of Pharmacy, Tongji Medical College, Huazhong University of Science and Technology, Wuhan 430030, People’s Republic of China*

**Supporting Information**

**Figure S1.** ^1^H-NMR spectrum of compound **1** (400 MHz in pyridine-*d*_5_)

**Figure S2.** ^13^C-NMR spectrum of compound **1** (100 MHz in pyridine-*d*_5_)

**Figure S3.** DEPT spectrum of compound **1**

**Figure S4.** HSQC spectrum of compound **1**

**Figure S5.** HMBC spectrum of compound **1**

**Figure S6.** ^1^H–^1^H COSY spectrum of compound **1**

**Figure S7.** NOESY spectrum of compound **1**

**Figure S8.** UV spectrum of compound **1**

**Figure S9.** IR spectrum of compound **1**

**Figure S10.** HRESIMS spectrum of compound **1**

**Figure S11.** ^1^H-NMR spectrum of compound **2** (400 MHz in pyridine-*d*_5_)

**Figure S12.** ^13^C-NMR spectrum of compound **2** (100 MHz in pyridine-*d*_5_)

**Figure S13.** DEPT spectrum of compound **2**

**Figure S14.** HSQC spectrum of compound **2**

**Figure S15.** HMBC spectrum of compound **2**

**Figure S16.** ^1^H–^1^H COSY spectrum of compound **2**

**Figure S17.** NOESY spectrum of compound **2**

**Figure S18.** UV spectrum of compound **2**

**Figure S19.** IR spectrum of compound **2**

**Figure S20.** HRESIMS spectrum of compound **2**

**Figure S21.** ^1^H-NMR spectrum of compound **3** (400 MHz in pyridine-*d*_5_)

**Figure S22.** ^13^C-NMR spectrum of compound **3** (100 MHz in pyridine-*d*_5_)

**Figure S23.** DEPT spectrum of compound **3**

**Figure S24.** HSQC spectrum of compound **3**

**Figure S25.** HMBC spectrum of compound **3**

**Figure S26.** ^1^H–^1^H COSY spectrum of compound **3**

**Figure S27.** NOESY spectrum of compound **3**

**Figure S28.** UV spectrum of compound **3**

**Figure S29.** IR spectrum of compound **3**

**Figure S30.** HRESIMS spectrum of compound **3**

**Figure S31.** The concentration-response data of cytotoxic effect on tumor cells of compounds **1**–**3**.

**
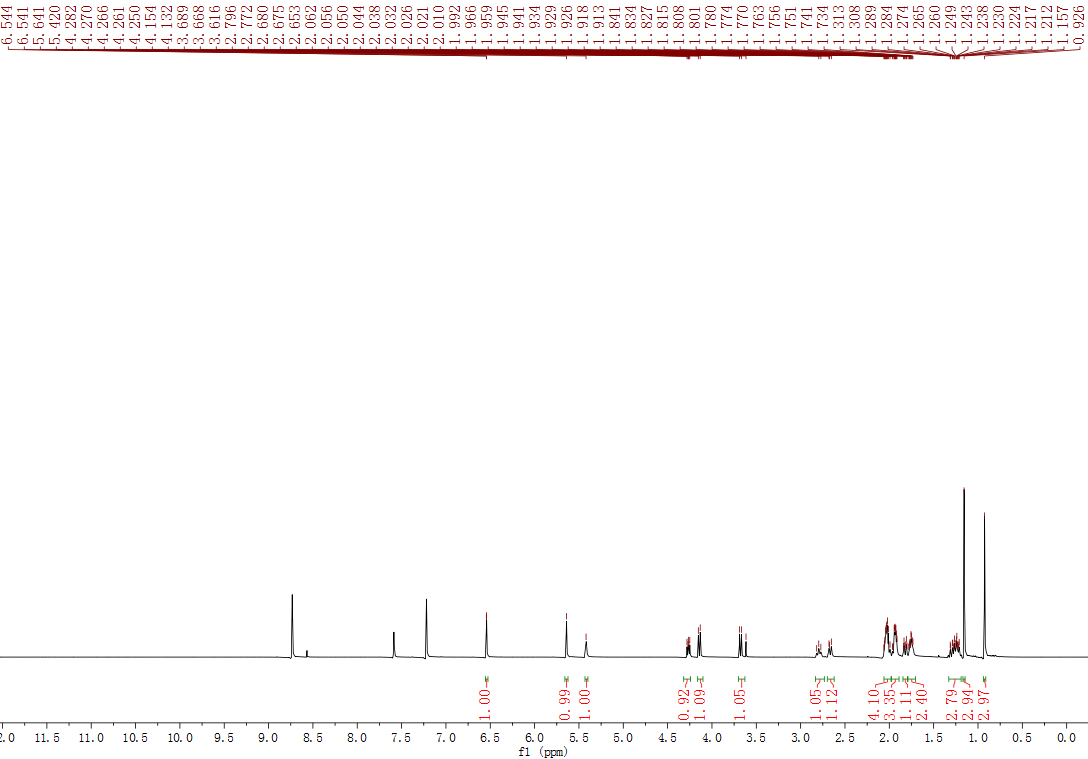
**

**Figure S1.** ^1^H-NMR spectrum of compound **1** (400 MHz in pyridine-*d*_5_)

**
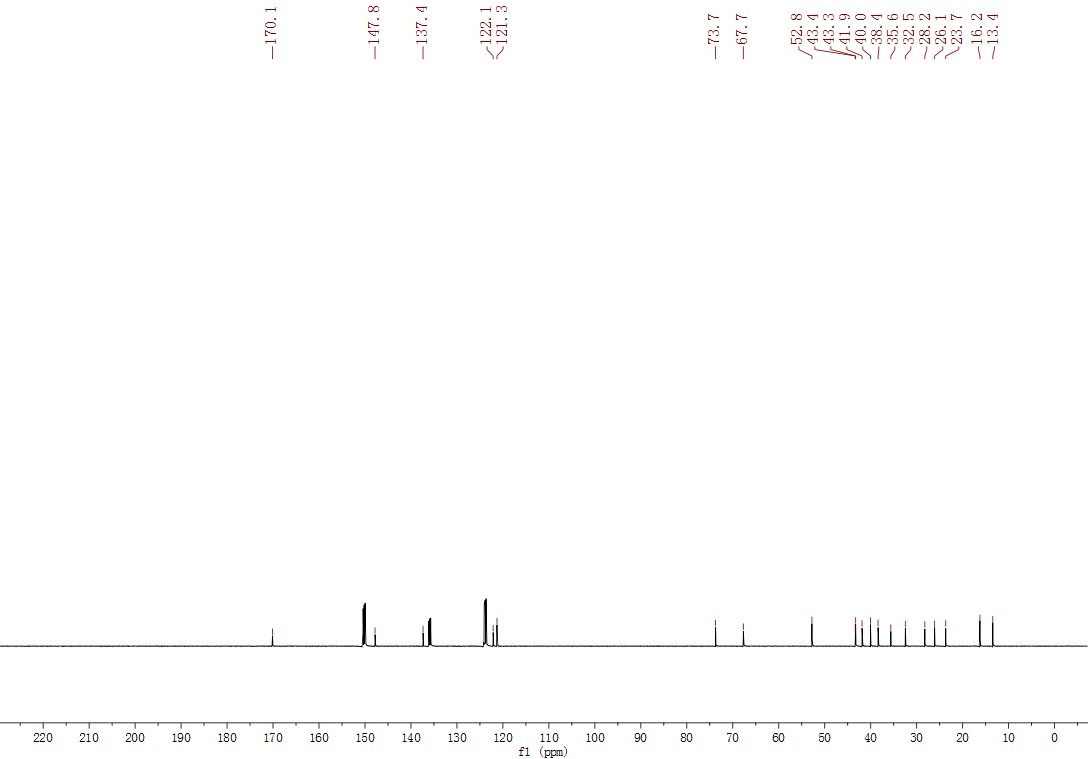
**

**Figure S2.** ^13^C-NMR spectrum of compound **1** (100 MHz in pyridine-*d*_5_)

**
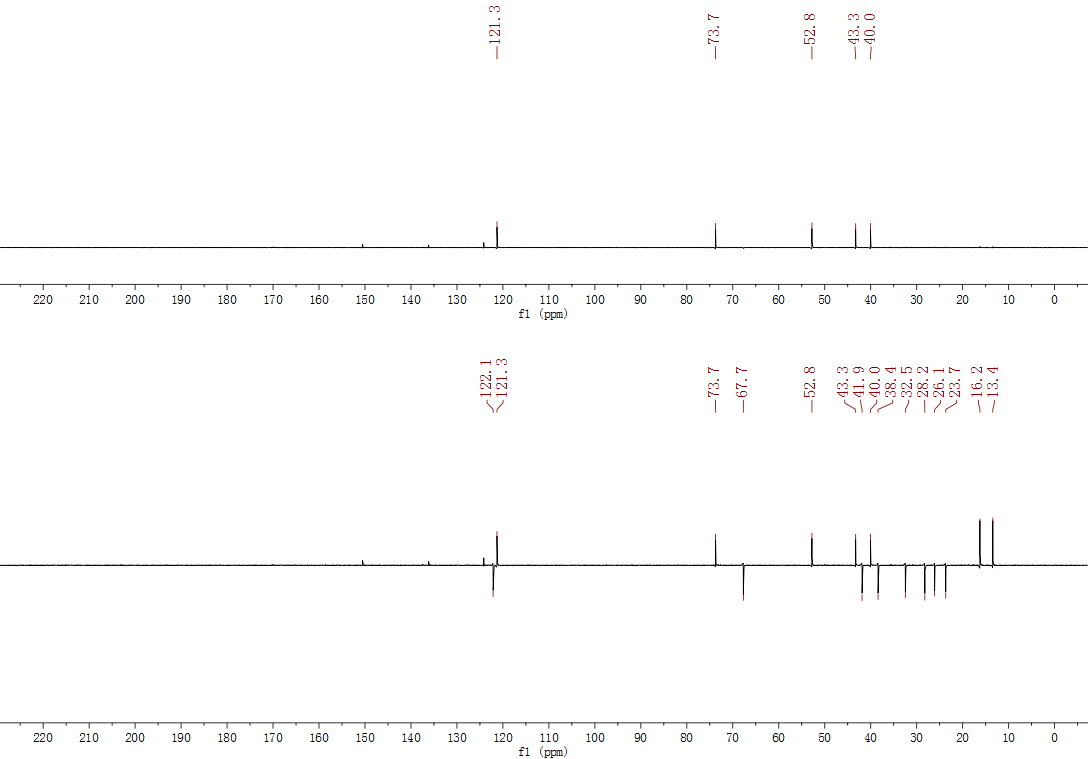
**

**Figure S3.** DEPT spectrum of compound **1**

**
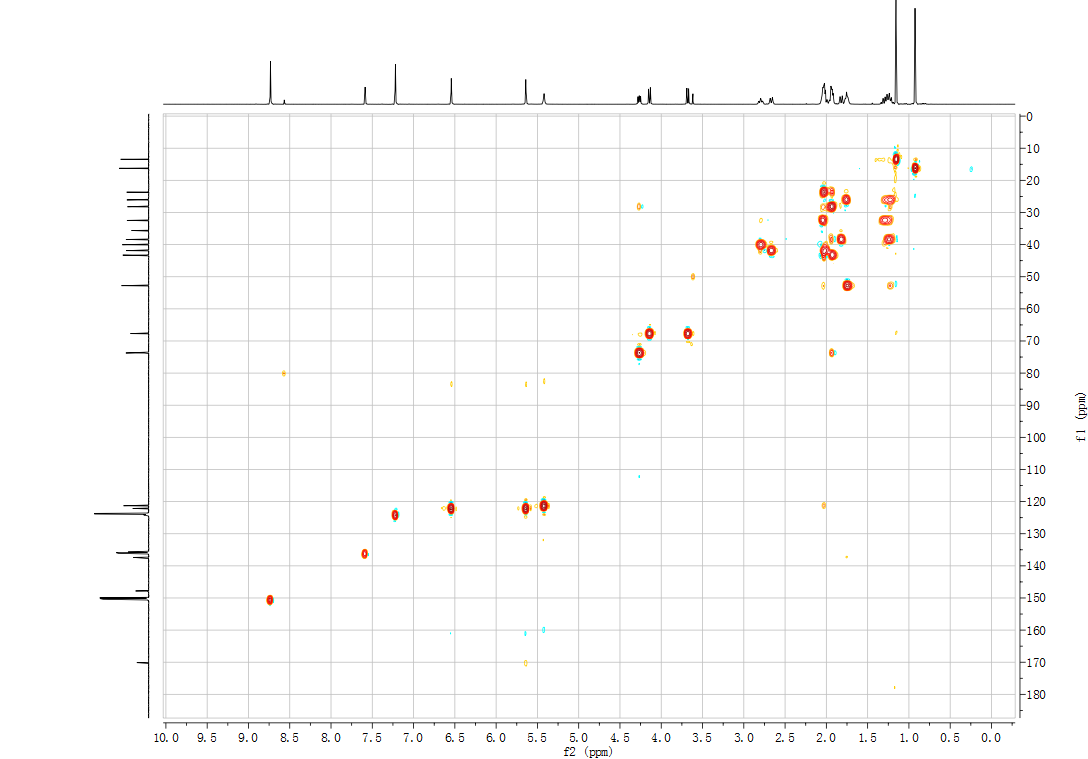
**

**Figure S4.** HSQC spectrum of compound **1**

**
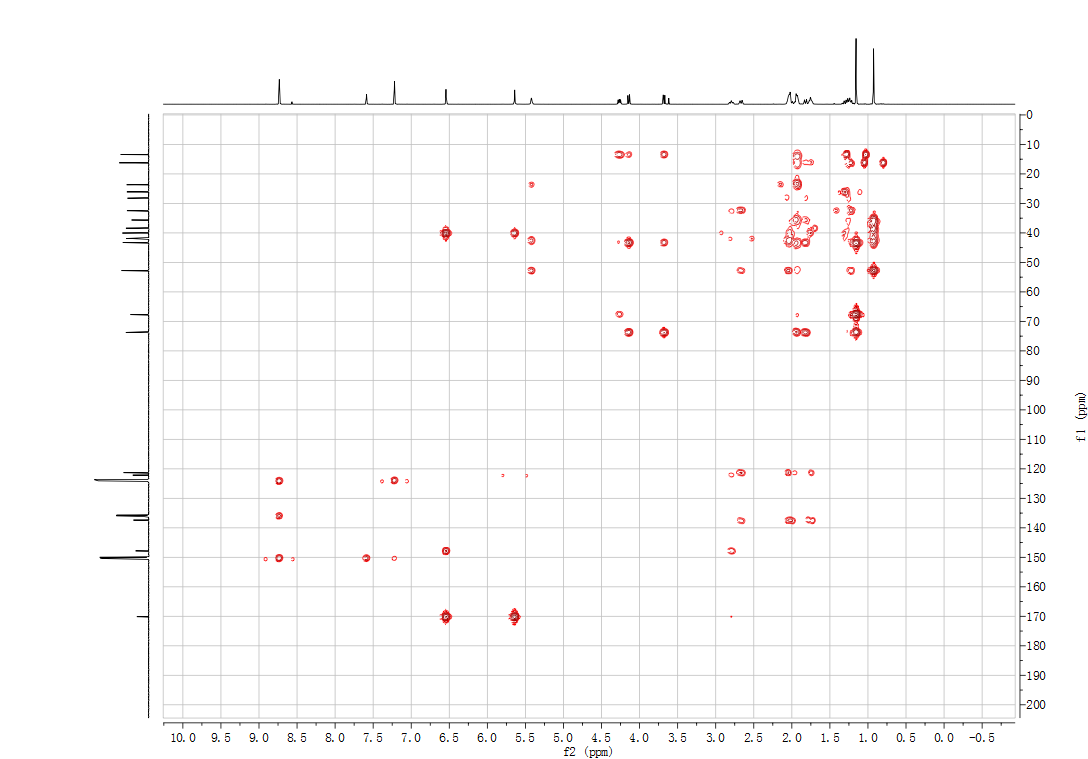
**

**Figure S5.** HMBC spectrum of compound **1**

**
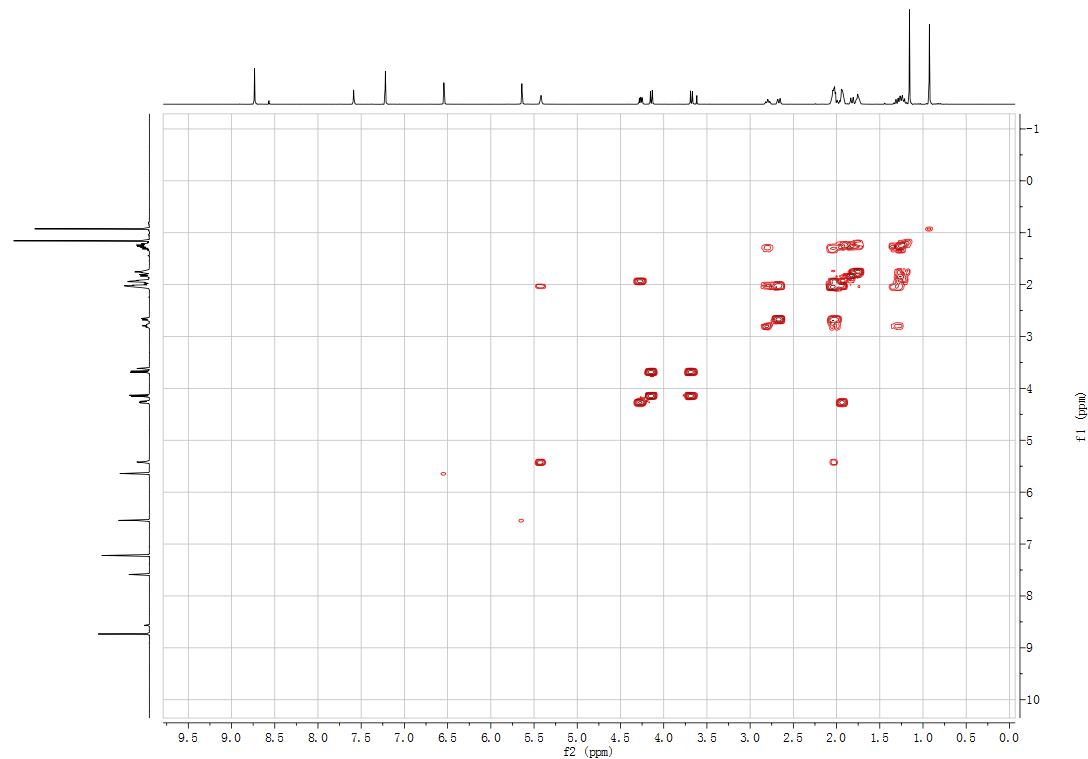
**

**Figure S6.** ^1^H–^1^H COSY spectrum of compound **1**

**
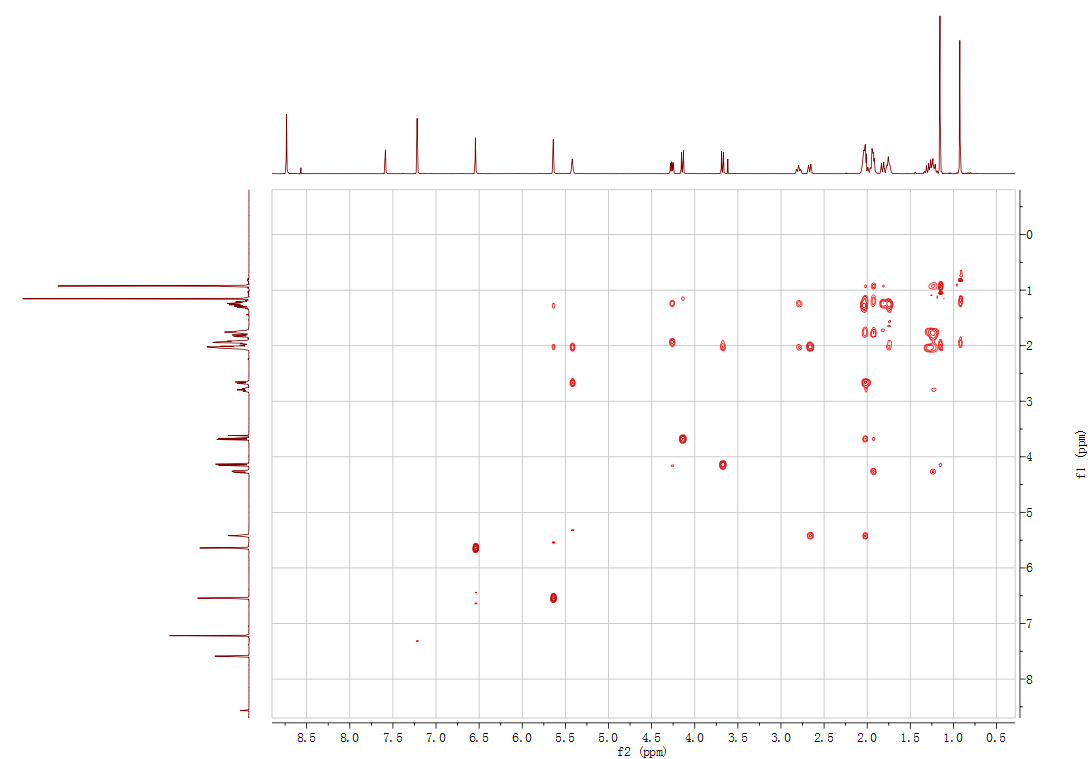
**

**Figure S7.** NOESY spectrum of compound **1**


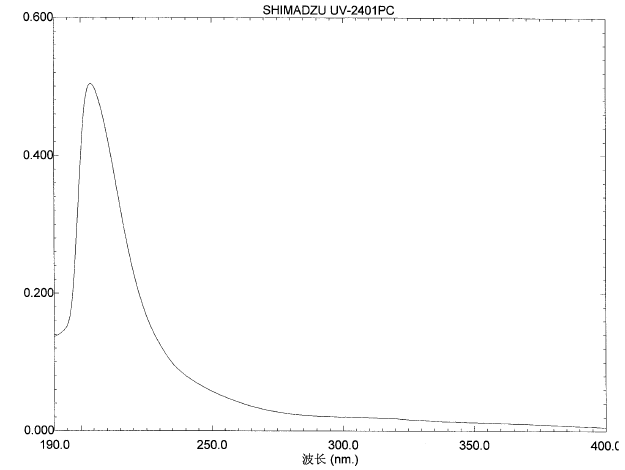


**Figure S8.** UV spectrum of compound **1**


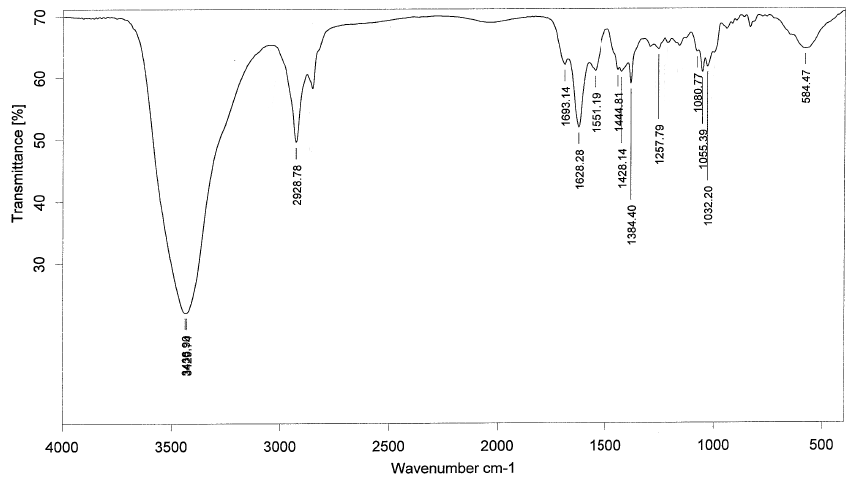


**Figure S9.** IR spectrum of compound **1**


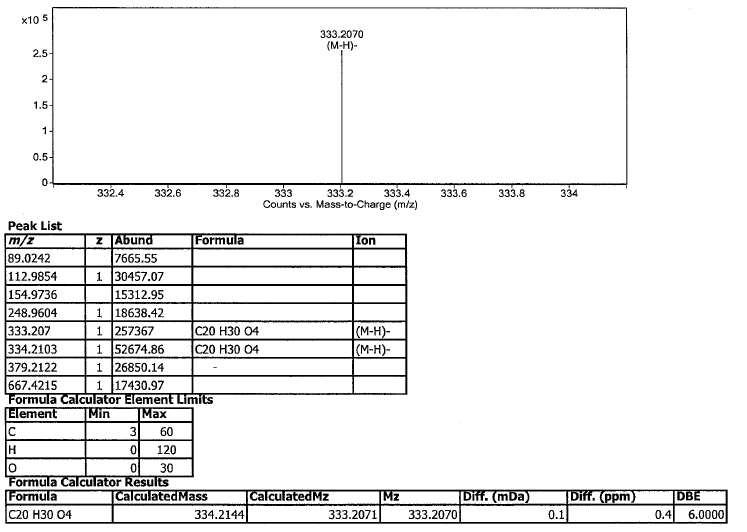


**Figure S10.** HRESIMS spectrum of compound **1**


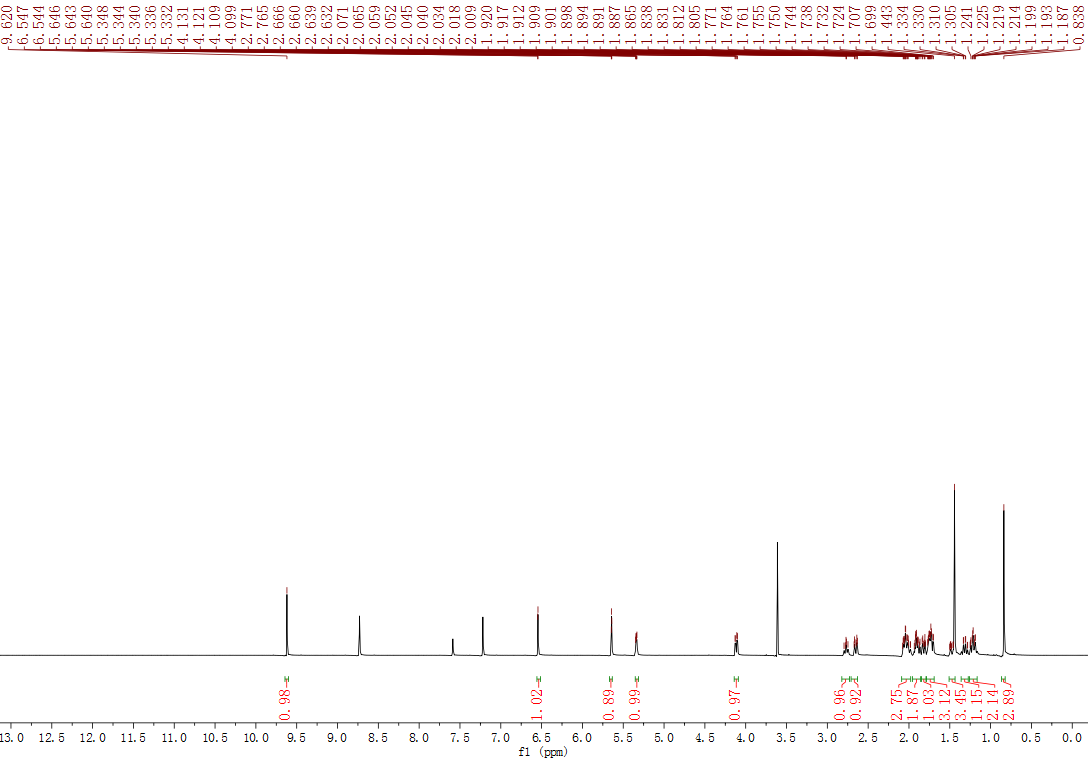


**Figure S11.** ^1^H-NMR spectrum of compound **2** (400 MHz in pyridine-*d*_5_)

**
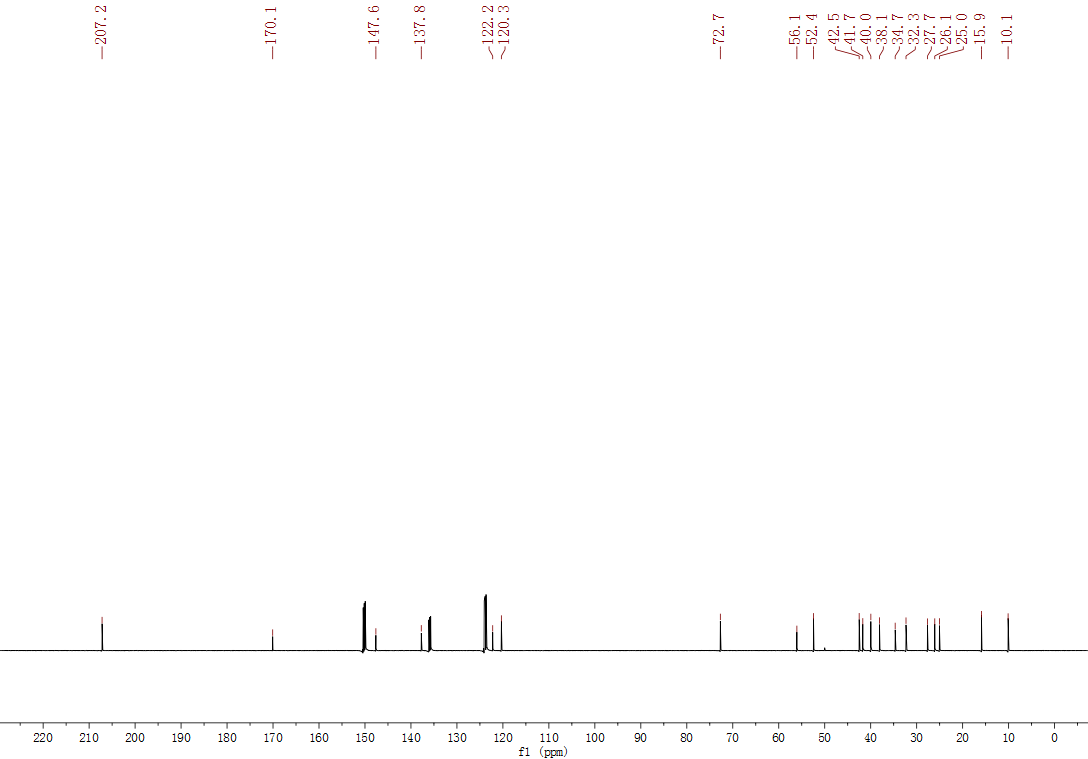
**

**Figure S12.** ^13^C-NMR spectrum of compound **2** (100 MHz in pyridine-*d*_5_)

**
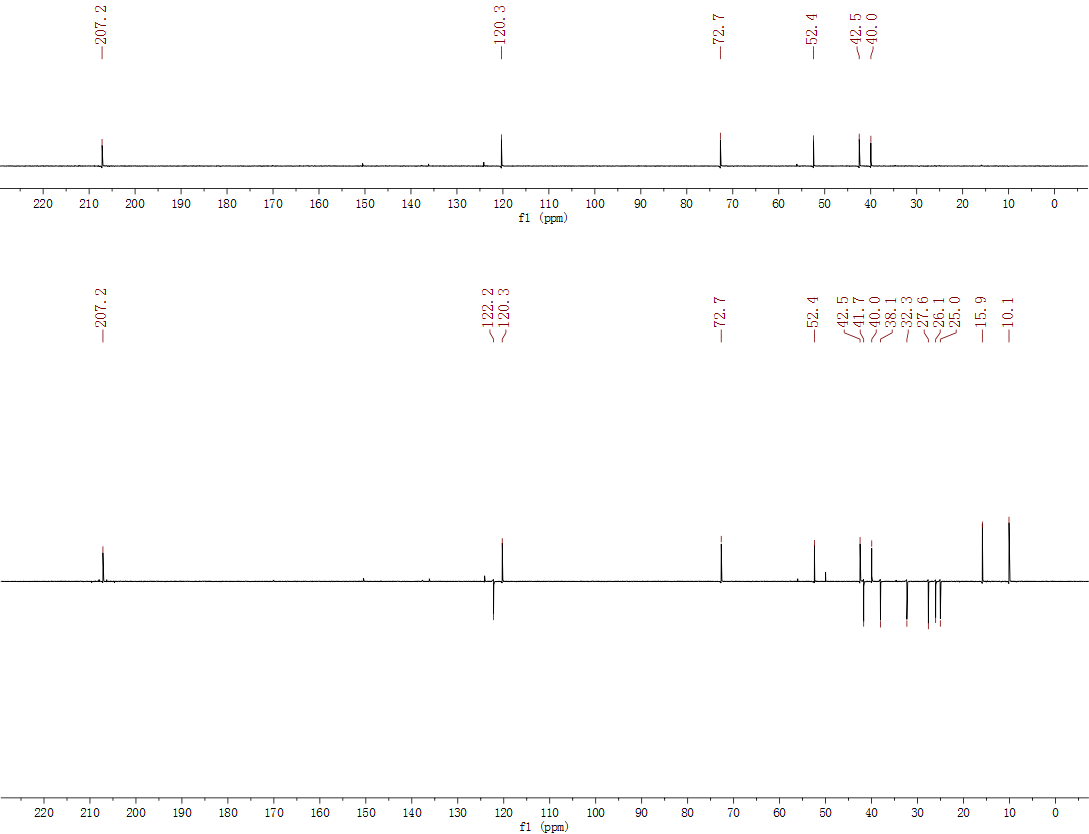
**

**Figure S13.** DEPT spectrum of compound **2**

**
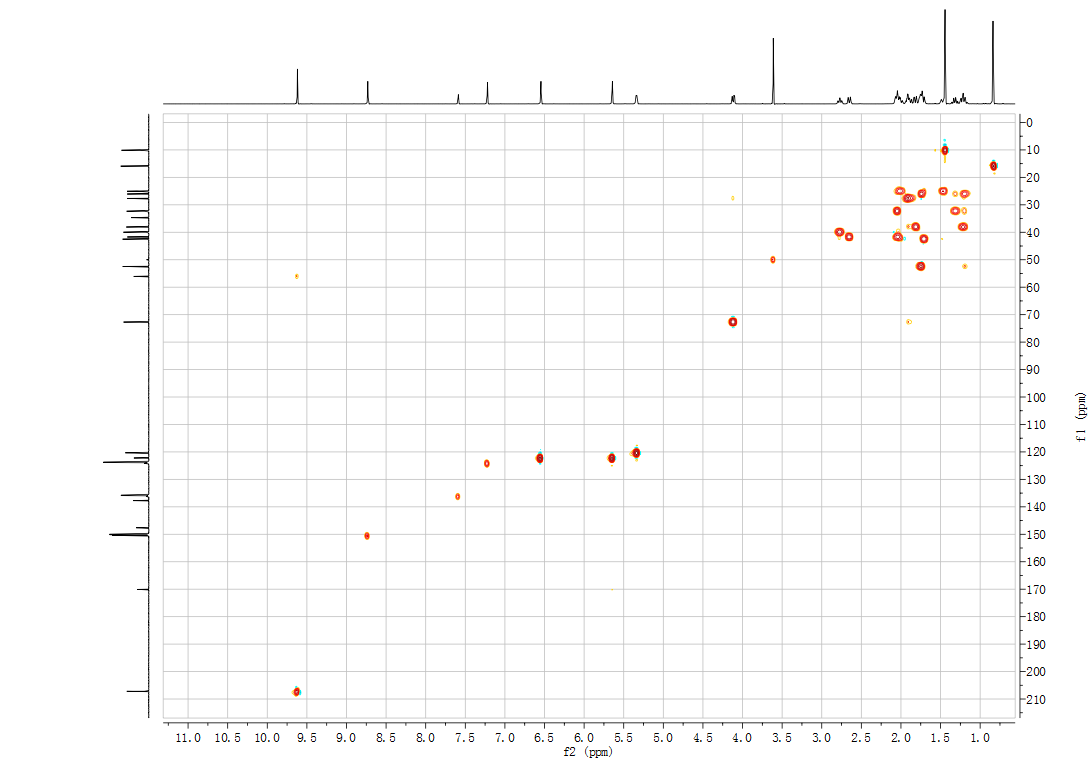
**

**Figure S14.** HSQC spectrum of compound **2**

**
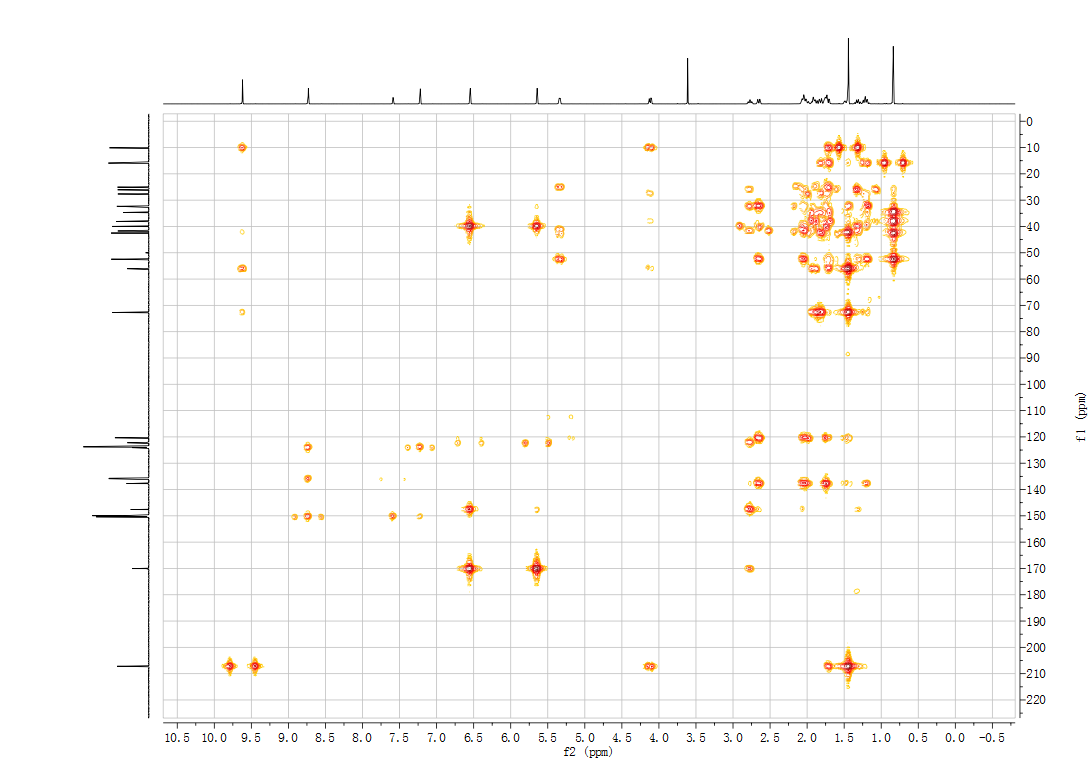
**

**Figure S15.** HMBC spectrum of compound **2**

**
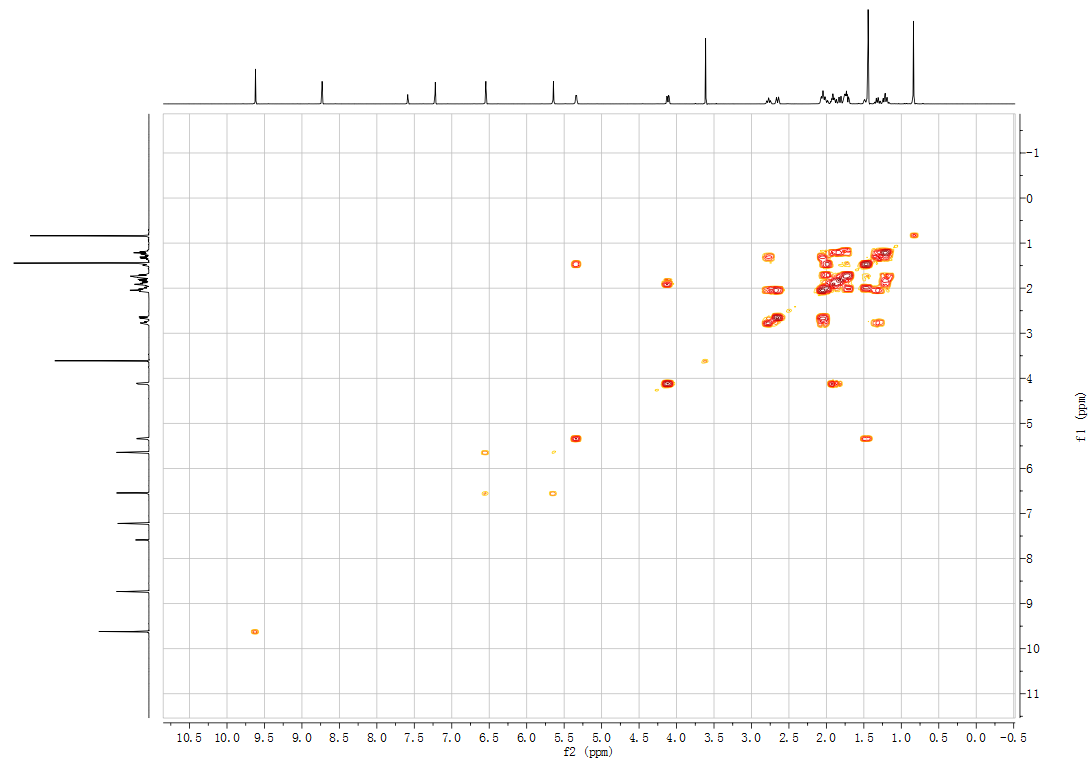
**

**Figure S16.** ^1^H–^1^H COSY spectrum of compound **2**

**
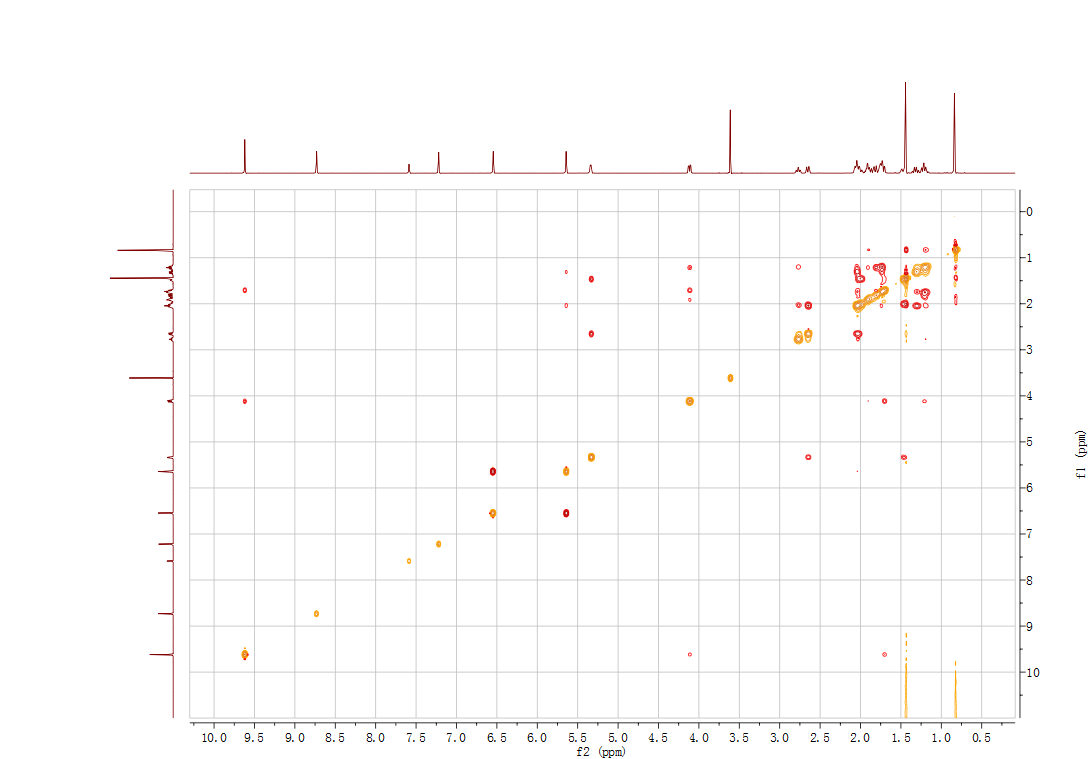
**

**Figure S17.** NOESY spectrum of compound **2**


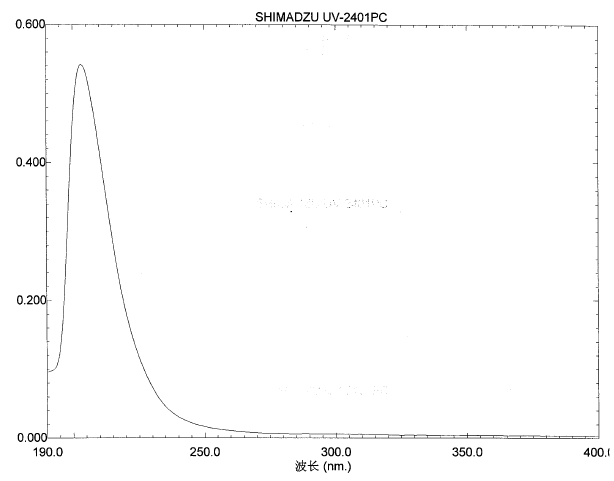


**Figure S18.** UV spectrum of compound **2**


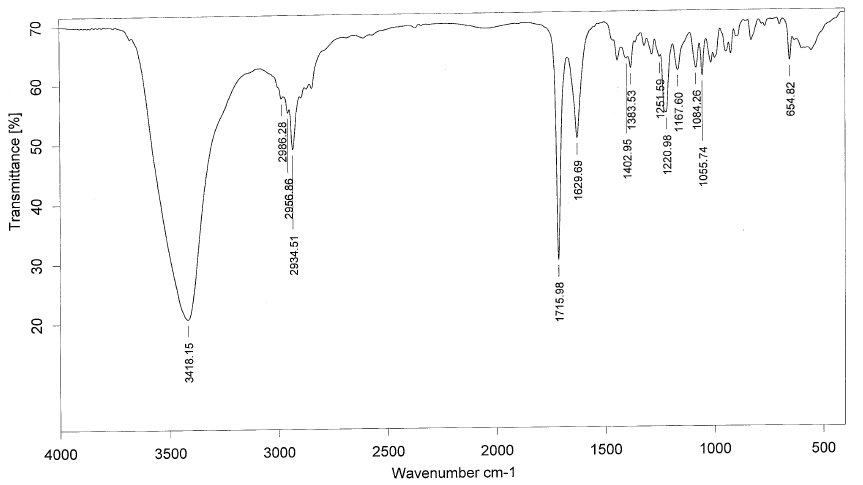


**Figure S19.** IR spectrum of compound **2**


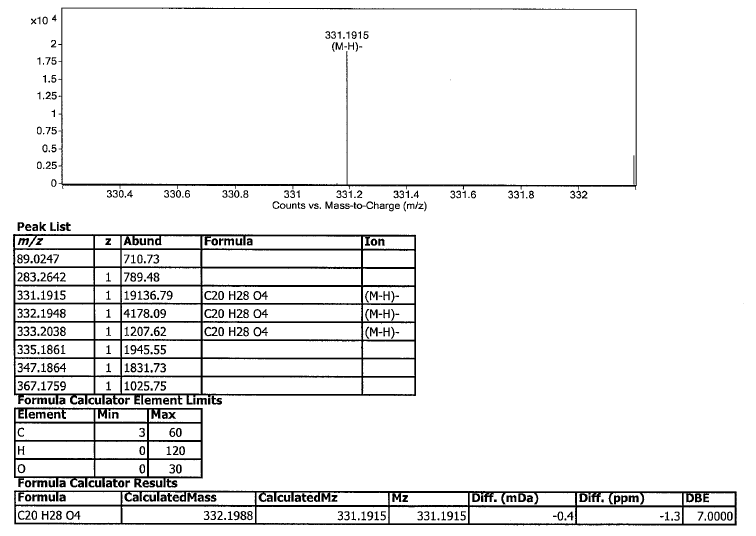


**Figure S20.** HRESIMS spectrum of compound **2**

**
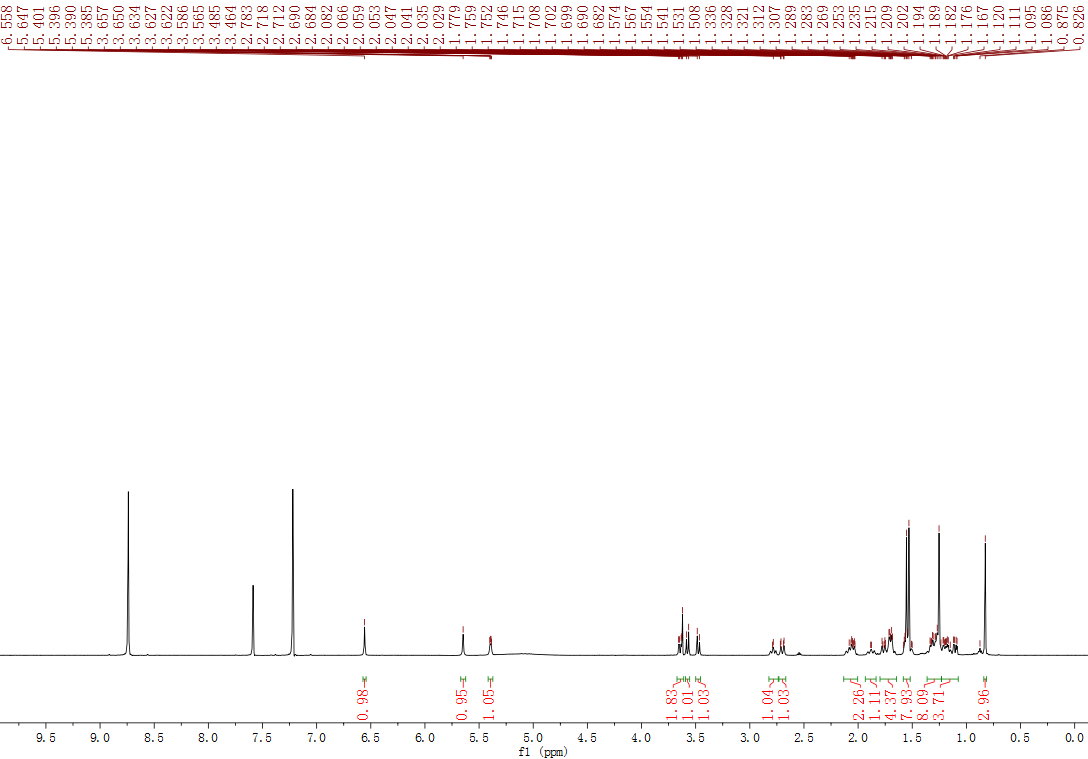
**

**Figure S21.** ^1^H-NMR spectrum of compound **3** (400 MHz in pyridine-*d*_5_)

**
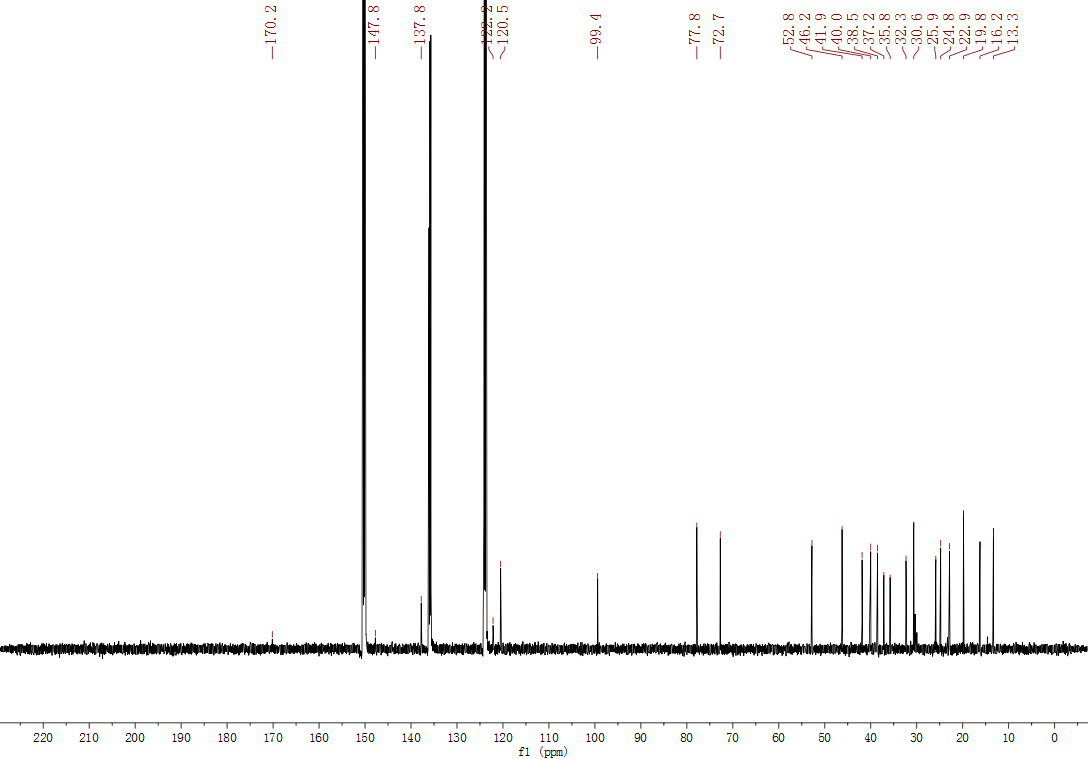
**

**Figure S22.** ^13^C-NMR spectrum of compound **3** (100 MHz in pyridine-*d*_5_)

**
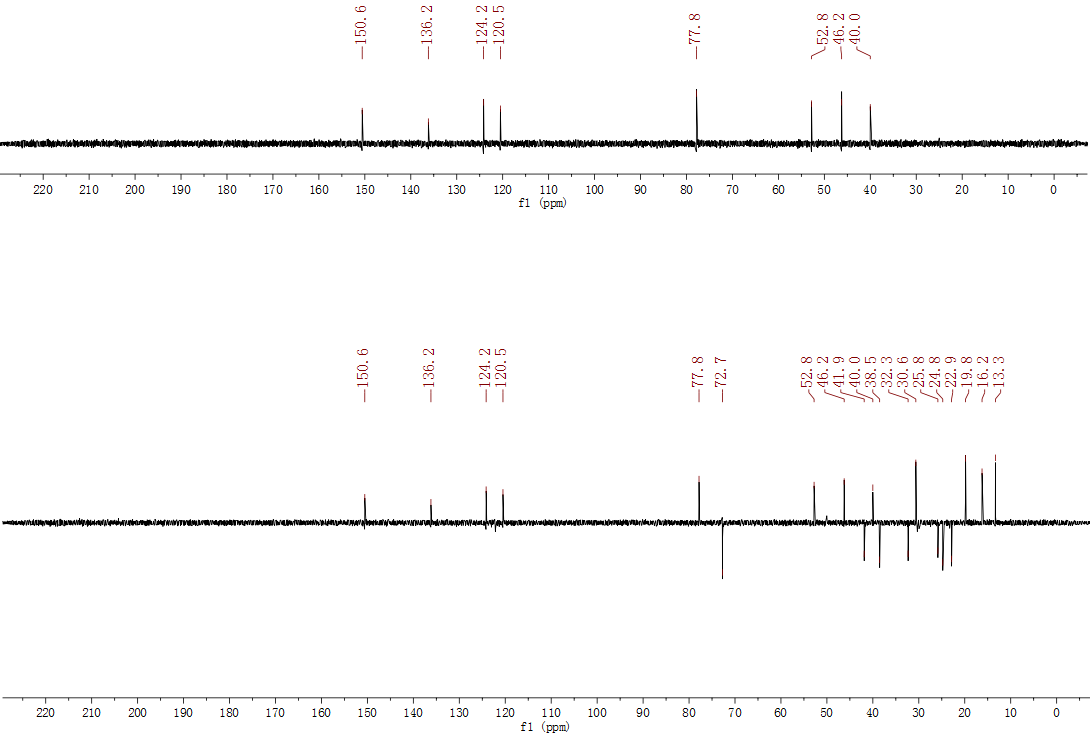
**

**Figure S23.** DEPT spectrum of compound **3**

**
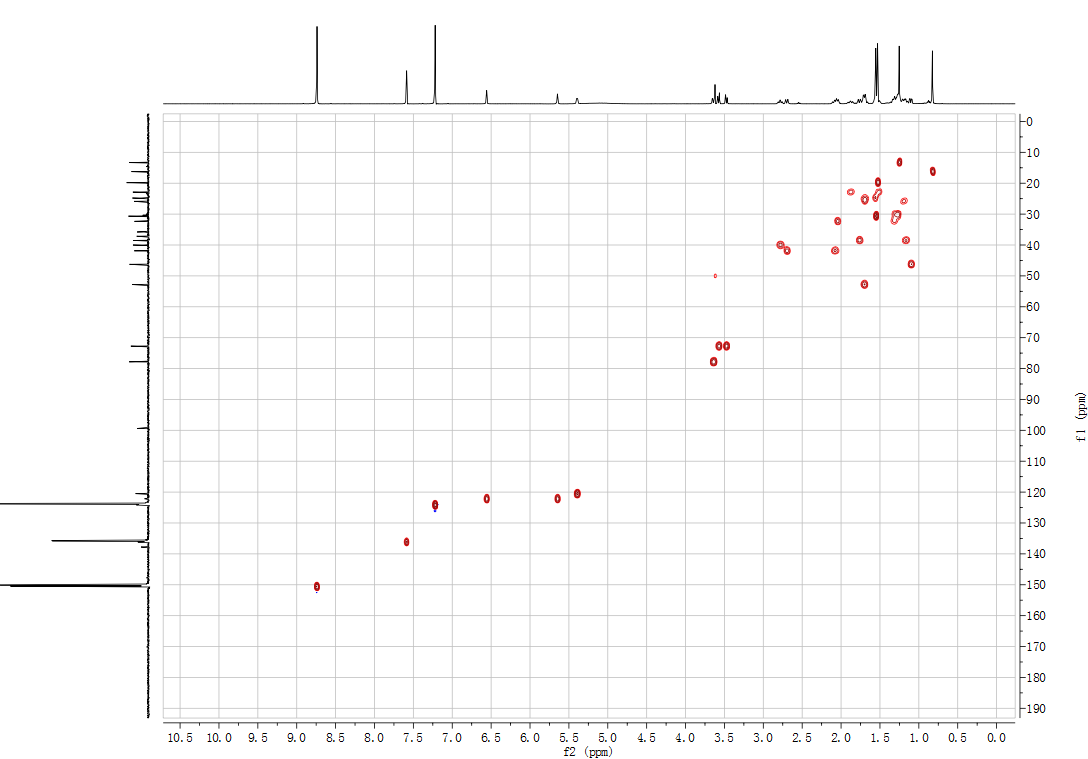
**

**Figure S24.** HSQC spectrum of compound **3**

**
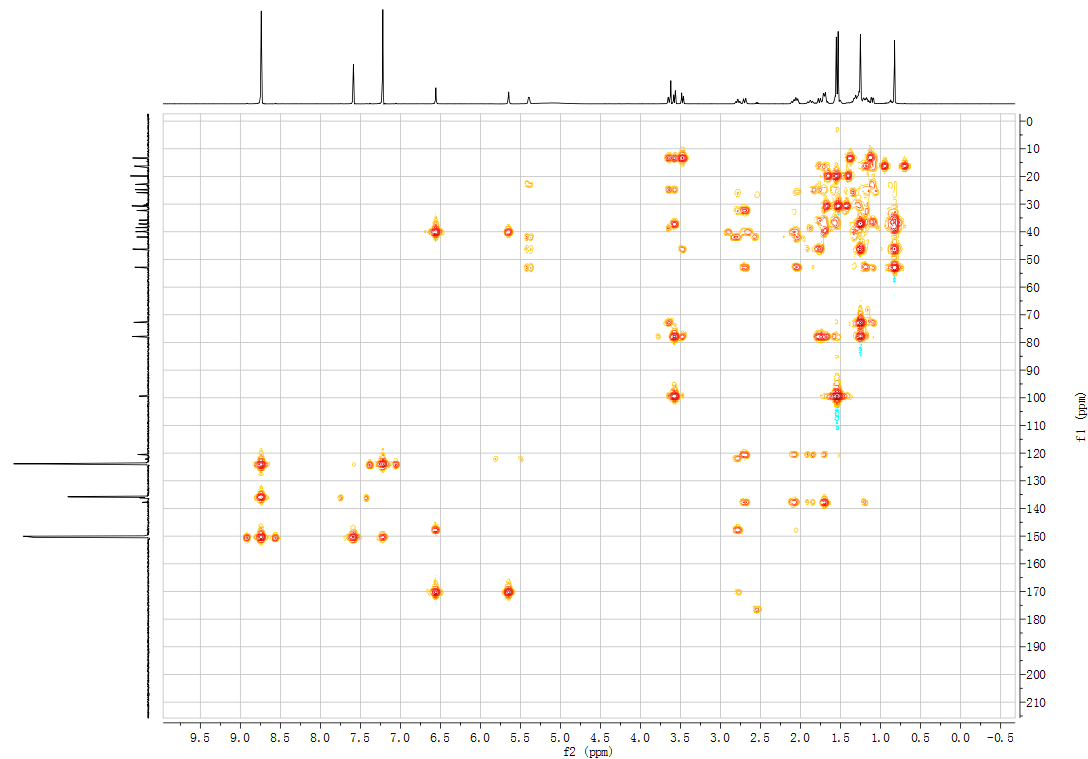
**

**Figure S25.** HMBC spectrum of compound **3**

**
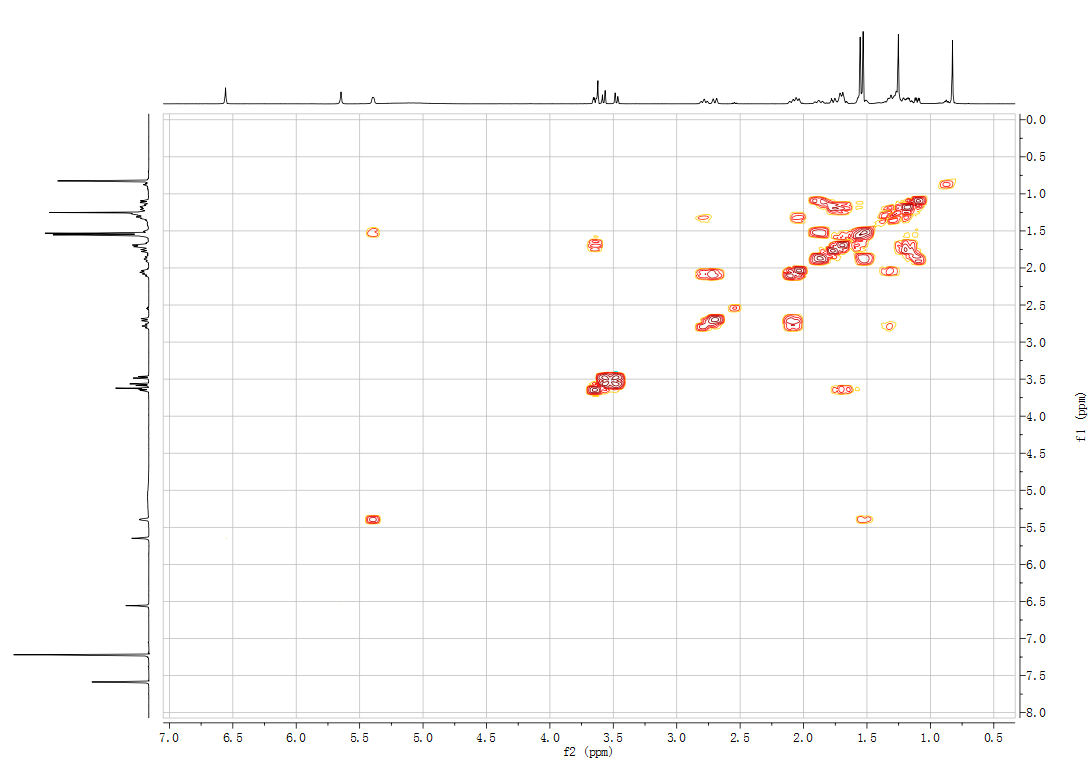
**

**Figure S26.** ^1^H–^1^H COSY spectrum of compound **3**

**
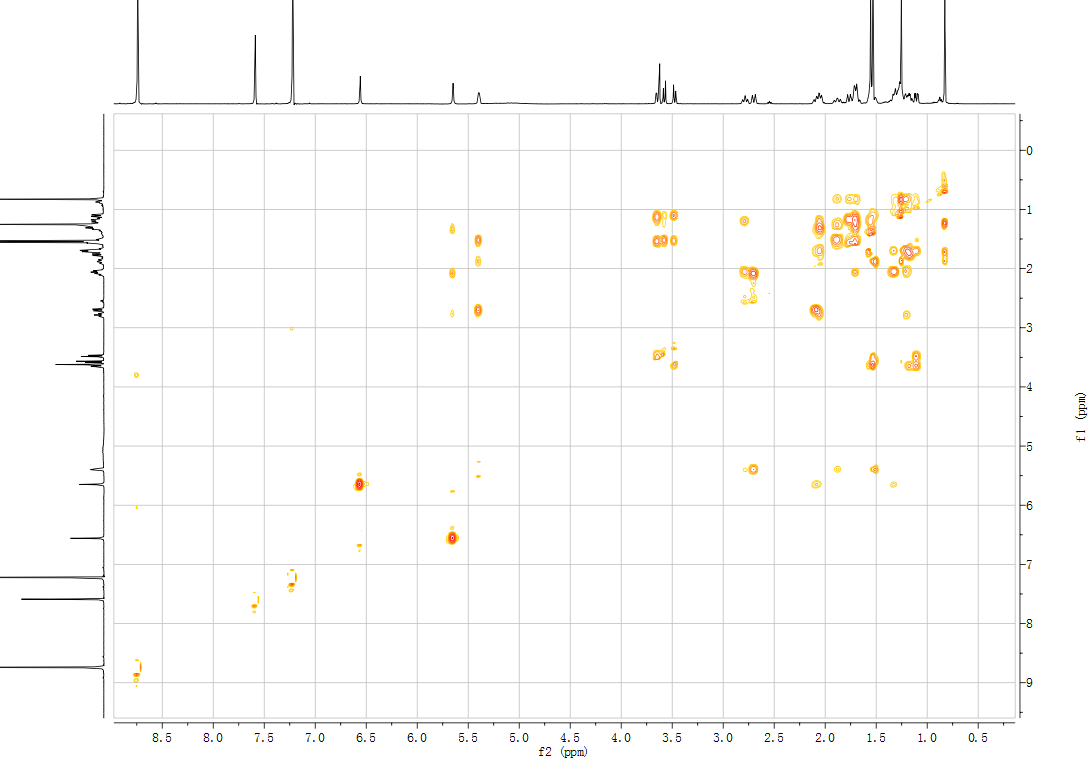
**

**Figure S27.** NOESY spectrum of compound **3**


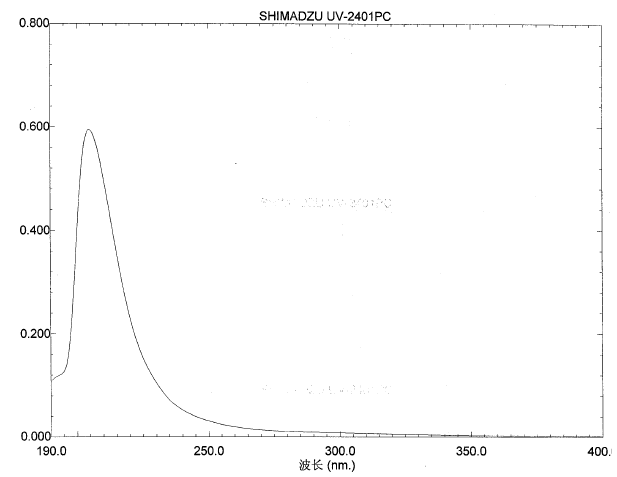


**Figure S28.** UV spectrum of compound **3**


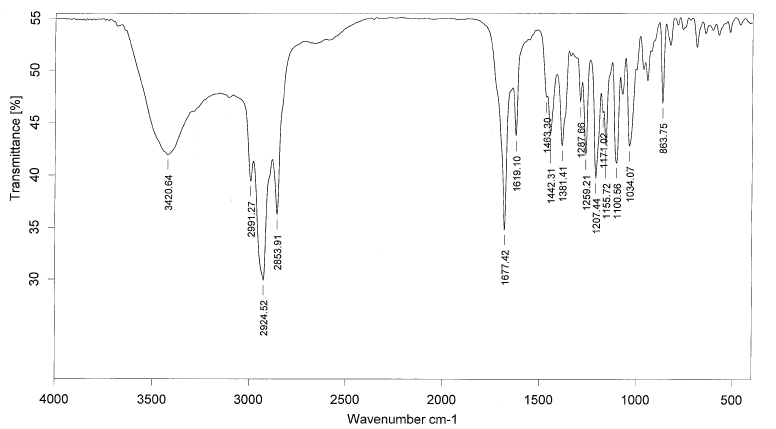


**Figure S29.** IR spectrum of compound **3**


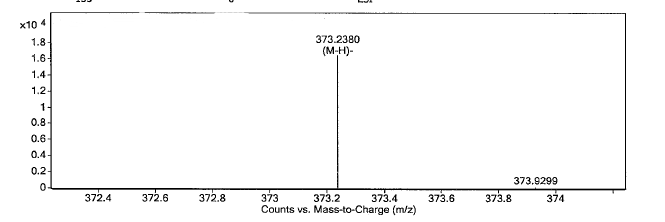


**Figure S30.** HRESIMS spectrum of compound **3**


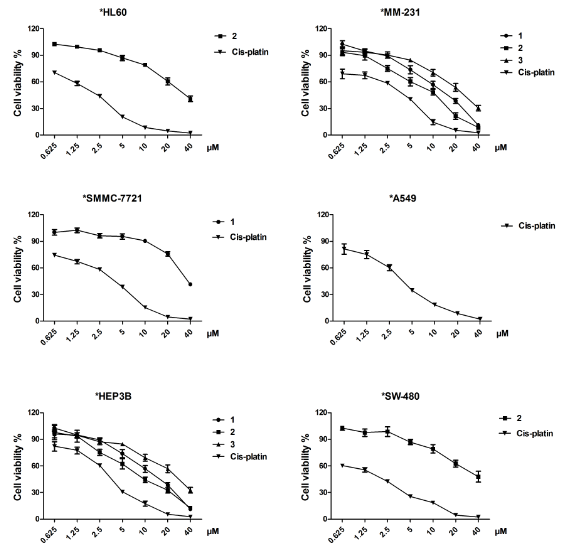
**Figure S31.** The concentration-response data of cytotoxic effect on tumor cells of compounds **1**–**3**.
